# Radiation-induced skin injury in the animal model of scleroderma: implications for post-radiotherapy fibrosis

**DOI:** 10.1186/1748-717X-3-40

**Published:** 2008-11-24

**Authors:** Sanath Kumar, Andrew Kolozsvary, Robert Kohl, Mei Lu, Stephen Brown, Jae Ho Kim

**Affiliations:** 1 Department of Radiation Oncology, Henry Ford Health System, Detroit, MI, USA; 2 Department of Biostatistics and Research Epidemiology, Henry Ford Health System, Detroit, MI, USA

## Abstract

**Background:**

Radiation therapy is generally contraindicated for cancer patients with collagen vascular diseases (CVD) such as scleroderma due to an increased risk of fibrosis. The tight skin (TSK) mouse has skin which, in some respects, mimics that of patients with scleroderma. The skin radiation response of TSK mice has not been previously reported. If TSK mice are shown to have radiation sensitive skin, they may prove to be a useful model to examine the mechanisms underlying skin radiation injury, protection, mitigation and treatment.

**Methods:**

The hind limbs of TSK and parental control C57BL/6 mice received a radiation exposure sufficient to cause approximately the same level of acute injury. Endpoints included skin damage scored using a non-linear, semi-quantitative scale and tissue fibrosis assessed by measuring passive leg extension. In addition, TGF-β1 cytokine levels were measured monthly in skin tissue.

**Results:**

Contrary to our expectations, TSK mice were more resistant (i.e. 20%) to radiation than parental control mice. Although acute skin reactions were similar in both mouse strains, radiation injury in TSK mice continued to decrease with time such that several months after radiation there was significantly less skin damage and leg contraction compared to C57BL/6 mice (p < 0.05). Consistent with the expected association of transforming growth factor beta-1 (TGF-β1) with late tissue injury, levels of the cytokine were significantly higher in the skin of the C57BL/6 mouse compared to TSK mouse at all time points (p < 0.05).

**Conclusion:**

TSK mice are not recommended as a model of scleroderma involving radiation injury. The genetic and molecular basis for reduced radiation injury observed in TSK mice warrants further investigation particularly to identify mechanisms capable of reducing tissue fibrosis after radiation injury.

## Background

Radiation fibrosis is frequently seen in patients undergoing high dose curative radiotherapy. It has been described in many tissues, including skin [1], lung [2]. Interestingly, collagen vascular disease (CVD) patients, particularly with scleroderma, are believed to be at increased risk of developing late complications of fibrosis after radiation therapy [3-6]. The increased toxicity is a serious clinical problem as many of these patients need radiation frequently as a part of cancer treatment and during breast conservation therapy for better cosmesis.

Cytokines, specifically Transforming growth beta 1 (TGF-β1) is considered to play a central role in mediating radiation induced tissue fibrosis [7]. Elevated levels of TGF-β1 have been associated with higher incidence of fibrosis after thoracic and abdomino-pelvic radiotherapy [8]. An abnormal increase in tissue TGF-β1 after radiation may underlie excessive fibrosis seen in CVD patients. Understanding the dynamics of TGF-β1 regulation after radiation in the setting CVD may be helpful in decreasing the long term toxicities associated with radiation therapy.

Tight skin (TSK) mouse has been proposed for use as an experimental animal model for scleroderma [9,10]. TSK mice display features of dermal fibrosis similar to those found in scleroderma [9]. The TSK phenotype results from duplication of a central portion of the fibrillin-1 gene [11]. Fibrillin-1 (FBN-1) is the major structural protein of connective tissue microfibrils that are key components of elastic fibers. The protein helps stabilize TGF-β in the extracellular matrix [12] and acts as an extracellular reservoir of growth factors [13]. TSK mutation leads to the production and secretion of a larger mutant FBN-1 protein [14]. The mutant protein expresses an increase in the number of TGF-β binding motifs resulting in more efficient binding of TGF-β [15]. Also the altered FBN-1 containing microfibrils become unstable and undergo proteolysis readily in comparison to wild-type FBN-1 [16].

Mutations in FBN-1 are associated with various connective tissue disorders in humans including Marfan syndrome (MFS) [17]. Abnormal expression of FBN-1 has also been noted in systemic sclerosis [18]. In the present study, we correlated the TGF-β1 levels with tissue injury and fibrosis seen after radiation in TSK mouse. The results would establish TSK mouse as an animal model for studying radiation induced fibrosis in the setting of scleroderma.

## Methods

15 mice aged five weeks were exposed to either two fractions of 30 Gy (TSK mice) or two fractions of 25 Gy (C57BL/6 mice) to the hind limbs. The damage to their skin was scored using a semi-quantitative scale. Tissue fibrosis was assessed by measuring passive leg extension.

### Mice

Male TSK/+ mice, and parental C57BL/6 pa/pa mice (+/+) were obtained from Jackson Laboratory (Bar Harbor, ME). TSK mice are black and are heterozygous for a dominant Fbn1Tsk mutation and a recessive Pldnpa mutation. There is no indication that the recessive Pldnpa mutation contributes to the phenotype of radiation damage.

All experiments performed in this study were approved by and in accordance with the guidelines of the Institutional Animal Care and Use Committee. The mice were kept in individual separate cages under specific pathogen free conditions before and throughout the experiments. This prevents any skin damage that might have been caused by the animals rather than radiation. The animals' health status was checked daily by the scientific investigators, the institutional animal care personnel and reviewed daily with the staff veterinarian. On recommendation by the staff veterinarian, animals were administered topical antibiotic and/or systemic analgesic (Buprenex).

### Radiation treatment

Mice were anesthetized with an intraperitoneal injection of ketamine (100 mg/kg) and xylazine (8 mg/kg). After ten minutes, the animals, as many as ten at a time, were positioned in a plexiglass jig that allowed radiation exposure of the right posterior leg. Shielding was provided with a square primary collimator (12 cm × 12 cm) and a circular secondary Cerrobend collimator (three 1/2 value layers). The dose rate from a 6 MV linear accelerator was 2.5 Gy/min, using 75 cm source to the surface distance. A 2.0 cm tissue equivalent bolus was used to bring the maximal dose to the skin surface. Dose was prescribed to the Dmax and mice received the fractionated schedule (24 hours apart) as indicated for each experiment. Doses were confirmed using micro-TLD dosimetry.

### Skin effects

Skin damage was assessed using a non-linear, semi-quantitative scale (Table 1) that is similar to previously reported acute skin damage animal models [19]. Two unblinded observers were also used to confirm the skin damage score. Skin damage was measured approximately weekly for 16 weeks.

**Table 1:** Semi-quantitative Skin damage scores

SCORE	SKIN CHANGES
1.0	No effect

1.5	Minimal erythema, mild dry skin

2.0	Moderate erythema, dry skin

2.5	Marked erythema, dry desquamation

3.0	Dry desquamation, minimal dry crusting

3.5	Dry desquamation, dry crusting, superficial minimal scabbing

4.0	Patchy moist desquamation, moderate scabbing

4.5	Confluent moist desquamation, ulcers, large deep scabs

5.0	Open wound, full thickness skin loss

5.5	Necrosis

### Tissue fibrosis

Skin and tissue fibrosis attributable to radiation injury was assessed adopting previously published solid tissue endpoints of damage [20]. At multiple time points from 60 days onward passive leg extension from heel to the medial aspect of the proximal leg (i.e. crotch) was measured with calipers. Skin damage and leg contraction was measured in the same mouse.

### TGF-β1 analysis

Quantitative estimation of TGF-β1 protein level in the skin tissue was done at 0, 30, 60 and 90 days both in radiated and control mice by enzyme-linked immunosorbent assay (ELISA) technique. Skin samples were weighed before being mixed with tissue lysis buffer containing 0.5% Triton X-100, 2 ug/ml Aprotinin in 1× PBS to reach a concentration of 40 mg of tissue/mL of buffer. After homogenization and centrifugation, the supernatant was withdrawn and stored at -80°C until analysis. Analysis was carried out at different time points after radiation for total TGF-β1, as well as for active TGF-β1, with commercially available kits (Promega, Madison, WI). Measurements of active and total amounts of TGF-β1 were performed in separate steps. The active fraction of TGF-β1 was assayed directly in the ELISA plate using the kits provided. For measuring the total amount of TGF-β1, additional samples were acidified to pH 3.0 using 1 mol/L HCl, followed by 15-min incubation at 22°C, resulting in activation of all TGF-β1. To neutralise samples, 1 mol/L NaOH was supplemented before application to the second ELISA plate, according to the manufacturer's instructions. The results were normalized to total protein content based on the method by Lowry using a commercial protein assay (Bio-Rad, Hercules, CA).

### Statistics

Primary tests of significance between TSK and C57BL/6 mice were made for skin injury and leg extension. Skin damage data were not normally distributed. In contrast, leg extension data were normally distributed. Consequently, the medians (and range) for skin damage and the mean (with standard error of the means) for leg extension measurements were employed. A nonparametric median test was applied to the skin damage data to determine the level of significance between TSK and C57BL/6 mice at each of the two radiation doses. A two-way ANOVA test was used for leg extension data to assess the significance between the groups. For TGFβ1 analysis, Student's t test was used to assess the difference between two groups.

## Results

### Radiation-induced Acute and Chronic Skin Reaction

**Figure 1:**
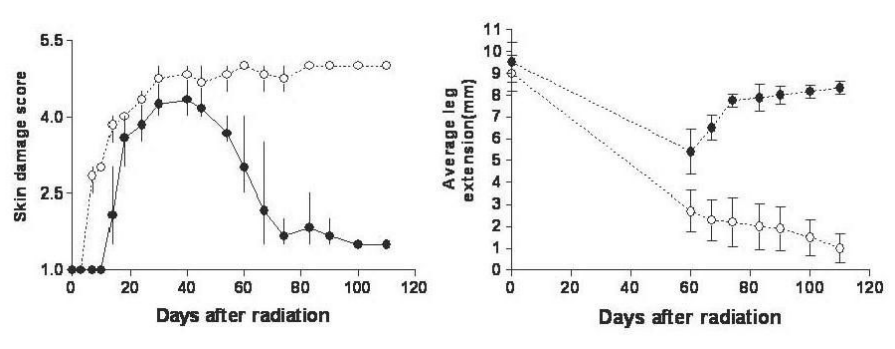
**Skin injury (panel A) and leg extension (panel B) in TSK (solid data points) and parental C57BL/6 mice (open data points) following 60 Gy (TSK) or 50 Gy (C57BL/6) given as two equal radiation fractions separated by 24 hours**. Each point for skin injury represents the median value for the group. The error bars represent minimum and maximum value of the range. Each point for leg extension data represents mean value for the group. The error bars represent the standard deviation of the mean.

Acute skin reactions were initially similar for the TSK and C57BL/6 parental mouse strains. For example, skin injuries up to six weeks following 60 Gy (2 fractions of 30 Gy separated by 24 hours) and 50 Gy (2 fractions of 25 Gy separated by 24 hours) were comparable in TSK and C57BL/6 strains respectively (Fig 1a). This translates into a radiation protection factor of 1.2 for TSK mouse. In sharp contrast to the acute response, at between two months and three months after radiation, a differential response to radiation in the two strains was evident with TSK mice showing less skin damage compared to C57BL/6 mice (p < 0.05) (Fig 1a). C57BL/6 mice received lower radiation dose compared to TSK mice as they tend to develop severe damage after two fractions of 30 Gy.

### Radiation-induced Leg Contraction

Measurements of radiation-induced leg contraction in TSK and C57BL/6 mice starting at two months and continuing to the end of the study paralleled the skin injury data. TSK mice receiving 30 Gy × 2 had significantly less leg contraction than C57BL/6 mice receiving 25 Gy × 2 (Fig 1b). The average leg extension at the end of the study period was 8.3 mm in TSK mice compared to an average leg extension of 1.0 mm in the C57BL/6 mice (p < 0.05) (Fig 1b). The average leg extensions at the same time in unirradiated TSK and C57BL/6 were 9.5 mm and 9 mm respectively. The implication is that there was significantly less fibrotic injury in TSK mice (Fig. 1b) compared to C57BL/6 mice.

### Analysis of TGF-β1 protein

**Figure 2:**
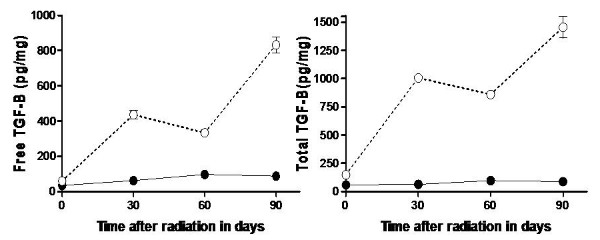
**Free (panel A) and total (panel B) transforming growth factor β1 (TGF-β1) measured in the skin tissue of TSK (solid data points) and C57BL/6 (open data points) mice following 60 Gy (TSK) or 50 Gy (C57BL/6) given as two equal radiation fractions separated by 24 hours**. Each point for the TGF-β1 protein represents the mean value for the group. The error bars represent the standard deviation of the mean.

The levels of both free and total TGF-β1 weren't statistically different in the five week old TSK and C57BL/6 mice before radiation. But at days 30,60 and 90 after radiation (Fig. 2), the quantity of both free and total TGF-β1 were significantly higher in the skin of C57BL/6 mice compared to TSK mice (p < 0.05). The TGF-β1 values correlated with the degree of skin injury and fibrosis seen at the end the study. The quantity of TGF-β1 in the skin of unirradiated C57/BL6 and TSK mice did not change significantly during this period (data not shown).

## Discussion

**Figure 3:**
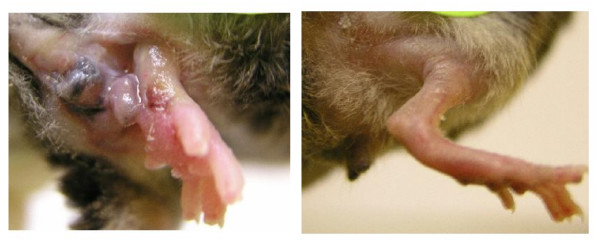
**Photograph of representative irradiated leg of TSK mice showing minor damage 110 days post-irradiation compared to parental control**. The TSK mice had relatively normal legs (panel B) post-irradiation except for hair loss whereas parental control mice (panel A) showed extensive skin and leg injuries following the radiation exposure.

Our results evidently demonstrate that TSK mice are resistant to radiation injury compared with the parental C57BL/6 strain with respect to the manifestation of late skin injury and fibrosis (Fig. 3). Even though both the TSK mice and control mice showed similar degrees of skin damage initially, the injury in TSK mice healed promptly and ultimately exhibited signs of less fibrosis. This study is the first report on the effects of radiation in an animal model for scleroderma.

Collagen vascular disease is clinically considered a relative contraindication for radiation therapy [21]. Scleroderma patients are statistically at higher risk for radiation induced complications in comparison to other collagen vascular disorders [4,5]. There have been reports of exaggerated cutaneous and internal fibrotic reaction following radiation therapy in scleroderma patients [3,22]. Consequently, the expectation of radiation response in an experimental model of scleroderma such as TSK mouse is for increased skin damage and fibrosis. But compared to parental C57BL/6 mice, TSK mice showed decreased radiation induced skin injury and fibrosis.

The underlying causes of the fibrotic disorder characteristic of unirradiated TSK mice have been a matter of debate. One probable scenario is based on the observations that increased TGF-β has an established association with increased fibrosis and that the mutated FBN-1 binds more TGF-β than the wild type [11]. In fact, deletion of TGF-β in the heterozygote TSK mouse resulted in less fibrosis [23]. It has been hypothesized that a breakdown of mutant FBN-1 containing unstable microfibrils could lead to a release of sequestered TGF-β which in turn could stimulate fibrosis [16]. Increased TGF-β activity secondary to abnormal FBN-1 leading to increased extracellular matrix deposition has also been hypothesized in initiating the fibrotic process [24].

The TGF-β family of proteins is synthesized as pro-proteins in association with latency associated peptide (LAP) which keeps the TGF-β in an inactive form [25]. The concentration of biologically active TGF-β is dependent on the conversion from its latent form which requires dissociation from LAP; a process termed latent TGF-β activation. The latent TGF-β binding protein (LTBP) binds latent TGF-β and helps it's targeting to the extracellular matrix [26]. The LTBP interacts with FBN-1 of the microfibrils and stabilize latent TGF-β in the extra cellular matrix [12]. Thus FBN-1 plays a critical role in the activation and signaling of TGF-β.

TGF-β has been implicated in the pathogenesis of diseases such as MFS [27] and scleroderma [28,29]. Recently, abnormal FBN-1 has been hypothesized to be the cause aberrant TGF-β signaling in scleroderma [24]. It seems that an altered FBN-1/TGF-β pathway is common to pathogenesis MFS, scleroderma and TSK mouse.

We measured the levels of TGF-β1 in irradiated TSK and C57BL/6 mice skin and correlated them with the level of tissue injury and fibrosis. Both free (active) and total TGF-β1 was higher in C57BL/6 mouse skin at all time points after radiation compared to the TSK mouse and correlated well with the higher degree of skin injury and fibrosis seen in C57BL/6 mouse after radiation. This seems counter intuitive as TSK mice were expected to show greater fibrosis after radiation due to their aberrant TGF-β1 signaling. Similar to TSK mouse, even though dysregulation of TGF-β1 activation secondary to mutation in FBN-1 is implicated in pathogenesis of MFS, patients with MFS apparently tolerate radiation treatment [30]. In contrast, scleroderma patients are known to be at risk of increased fibrosis after radiation therapy.

There may be several possible reasons for the observed results. Tight binding of TGF-β1/LTBP to the abnormal FBN-1 may result in decreased release of the biologically active TGF-β1 in TSK mice after tissue injury. Indeed, radiation is known to induce activation of latent TGF-β1 to its active form *in vivo *[31]. This seems not to be the case as we observed lower levels of both free and bound TGF-β1 (after acid activation) in the TSK mouse skin compared to C57BL/6 mouse. Alternatively, breakdown of unstable microfibrils could lead depletion of TGF-β1 stores and may blunt TGF-β1 mediated effects including fibrosis after radiation injury. The heterozygote TSK/+ mouse also produces comparable amounts of normal FBN-1 molecule along with larger abnormal FBN-1 molecule [14]. But this doesn't seem to increase the local TGF-β1 availability after radiation injury in TSK mice. It may also be that the abnormal FBN-1 molecule protects TSK mice from radiation induced skin injury by a mechanism not involving TGF-β1.

## Conclusion

Based on the data presented, we conclude that the TSK mouse is not a suitable model to study the effects of radiation in case of scleroderma. Further studies are required to elucidate the role of FBN-1 in controlling TGF-β1 signaling in TSK mouse. The underlying mechanism of radiation resistance in TSK mouse can be exploited to prevent long term fibrosis in patients undergoing radiation therapy.

## Abbreviations

ANOVA: analysis of variance; ELISA: enzyme-linked immunosorbent assay; FBN-1: fibrillin-1; LTBP: TGF-β1 binding protein; LAP: latency associated peptide; TGF-β1: transforming growth beta 1; TSK: tight skin mouse.

## Competing interests

The authors declare that they have no competing interests.

## Authors' contributions

SK designed and performed experiments, analyzed data and wrote the manuscript. AK and RK performed experiments. ML developed analytical tools. SB designed experiments, supervised its analysis and edited the manuscript. JHK designed experiments, supervised its analysis and edited the manuscript.
